# An AI model of sonographer’s evaluation+ S-Detect + elastography + clinical information improves the preoperative identification of benign and malignant breast masses

**DOI:** 10.3389/fonc.2022.1022441

**Published:** 2022-11-11

**Authors:** Pengfei Sun, Ying Feng, Chen Chen, Andre Dekker, Linxue Qian, Zhixiang Wang, Jun Guo

**Affiliations:** ^1^ Department of Ultrasound, Beijing Friendship Hospital, Capital Medical University, Beijing, China; ^2^ Department of Radiation Oncology (Maastro), GROW-School for Oncology and Reproduction, Maastricht University Medical Centre, Maastricht, Netherlands; ^3^ Department of Ultrasound, Aerospace Center Hospital, Beijing, China

**Keywords:** breast tumor, computer-aided diagnosis, ultrasonography, AI, radio frequency, diagnosis

## Abstract

**Purpose:**

The purpose of the study was to build an AI model with selected preoperative clinical features to further improve the accuracy of the assessment of benign and malignant breast nodules.

**Methods:**

Patients who underwent ultrasound, strain elastography, and S-Detect before ultrasound-guided biopsy or surgical excision were enrolled. The diagnosis model was built using a logistic regression model. The diagnostic performances of different models were evaluated and compared.

**Results:**

A total of 179 lesions (101 benign and 78 malignant) were included. The whole dataset consisted of a training set (145 patients) and an independent test set (34 patients). The AI models constructed based on clinical features, ultrasound features, and strain elastography to predict and classify benign and malignant breast nodules had ROC AUCs of 0.87, 0.81, and 0.79 in the test set. The AUCs of the sonographer and S-Detect were 0.75 and 0.82, respectively, in the test set. The AUC of the combined AI model with the best performance was 0.89 in the test set. The combined AI model showed a better specificity of 0.92 than the other models. The sonographer’s assessment showed better sensitivity (0.97 in the test set).

**Conclusion:**

The combined AI model could improve the preoperative identification of benign and malignant breast masses and may reduce unnecessary breast biopsies.

## Introduction

Breast cancer is one of the most common malignant tumors in women (1), and its morbidity and mortality are increasing yearly. Early screening, diagnosis, and timely treatment can effectively reduce mortality from breast cancer (2).

Ultrasound is an important modality for screening and diagnosing breast cancer (3). In addition to grayscale ultrasound, Doppler and elastography (4) are helpful for obtaining more diagnostic information, but their accuracy tends to be very operator-dependent. Especially when the benign and malignant features are not typical, it is difficult to give an objective and appropriate diagnosis in a timely manner (5, 6). Therefore, it is important to develop approaches to minimize the differences between sonographers’ diagnoses and thus improve the standardization, quantification, and accuracy of ultrasonography.

With the improvements in medical imaging and computer technology, computer-aided diagnosis is expected to address the above problems. Especially in medical imaging analyses, the computer can extract features that are invisible to human eyes. This makes the computer a powerful tool for aiding doctors in clinical diagnoses, such as Coronavirus (COVID-19) detection (7–13) and cancer diagnosis (14–16). AI also helps in the diagnosis of breast lesions and is an active field of research (17). For example, Md. Milon Islam proposed a machine-learning algorithm to predict breast cancer (18–20). S-Detect has implemented deep learning technology from the field of artificial intelligence (AI). This software can automatically identify the boundary and shape of breast masses and analyze and interpret grayscale ultrasound images. Some studies have confirmed that this technology has high diagnostic performance (21, 22). However, the factor of calcification is not taken into account in S-Detect, and it is very important in distinguishing benign from malignant breast nodules. Moreover, S-Detect has not been widely applied in clinical practice at present.

The diversity and complexity of breast cancer on sonographic images require more comprehensive information to make an accurate diagnosis. Therefore, it is proposed that an AI using a combination of information from a sonographer’s evaluation, S-Detect, elastography, and clinical characteristics could achieve high diagnostic accuracy. The purpose of this study was to build an AI model with preoperative clinical features to further improve the accuracy of the assessment of benign and malignant breast nodules. It is our hypothesis that an AI model combining the sonographer’s evaluation + S-Detect + elastography + clinical information could improve diagnostic performance and may reduce unnecessary breast biopsies.

## Materials and methods

### Study objective

Patients who underwent breast examination or puncture at the Beijing Friendship Hospital from March 2022 to July 2022 were selected. There were 145 patients in the training set and 34 patients in the independent test set. All patients underwent routine ultrasound, S-Detect, and strain elastography. According to the timing of their examination, all patients were divided into a training set and a test set. The inclusion criteria were as follows (1): female patients over 18 years old (2); complete ultrasound data; and (3) breast lesions classified as BI-RADS 3–5. Exclusion criteria (1): nonmass lesions that are difficult to detect by conventional ultrasound (2); cystic and polycystic lesions with mixed echogenicity (3); pregnancy or lactation, patients with an artificial prosthesis (4); patients undergoing neoadjuvant chemoradiotherapy; and (5) needle biopsy performed before ultrasonography. All patients signed informed consent forms before the examination.

### Ethics statements

This study is a prospective study. The study protocol was reviewed and approved by the institutional review board of our hospital. All patients and their families provided written informed consent.

### Imaging analysis

#### Ultrasound, S-Detect, elastography examinations, and clinical information collection

##### Ultrasound examinations

Ultrasound examinations were conducted using a 3–12 linear probe (RS80A with Prestige, Samsung Medison, Co., Ltd., Seoul, South Korea). A radiologist with 10 years of experience in breast imaging performed bilateral breast ultrasound examinations under the breast parameters. Bilateral whole-breast examinations were routinely performed. When a mass was detected, the location, size, shape, aspect ratio, edge, capsule, internal echo, calcification, and color Doppler US were recorded and evaluated. The radiologist made a judgment on breast lesions according to the fourth edition of the Breast Imaging Reporting and Data System (BI-RADS) (11).

##### S-Detect examinations

Additionally, the radiologist performed a computer-aided diagnosis (CAD) examination with S-Detect software. After entering S-Detect mode, the software automatically contours the lesion area. The radiologist manually corrected it if necessary. S-Detect automatically analyzes the ultrasound features according to the ultrasound BI-RADS lexicon. S-Detect selected the most appropriate criterion in each of the following categories: shape, orientation, margins, lesion boundaries, posterior features, and echo pattern. The final evaluation of the breast lesions by S-Detect was divided into two categories: possibly benign and possibly malignant. The S-Detect automatic analysis program was activated to output the S-Detect report.

##### Strain elastography

During the strain elastography examination, the areas of interest, including the lesions and the surrounding normal breast tissues, were imaged. The probe was held vertically to the chest, and it vibrated the breast tissue. When the pressure reached the ideal state, the compression guide bar turned green on the right side of the elastography interface. Green represents tissue deformation, and blue represents invisible tissue changes. Elastic scoring was performed for each nodule according to the standard method (23). Meanwhile, during the process of elastic mode, the E ratio (strain rate of the lesion/strain rate of the surrounding glandular tissue) and E-breast (strain rate of the breast lesion/strain rate of the surrounding fat) were calculated (4). Each lesion was measured three times, and the average value was taken.

##### Image interpretation

According to the ultrasound characteristics of the lesions, the breast lesions were classified into categories 3, 4, and 5 based on the BI-RADS lexicon by the same radiologist. The classification results were evaluated by a dichotomous method: BI-RADS 3 was “possibly benign;” BI-RADS 4 and 5 were “possibly malignant.”

### Statistical analyses

SPSS 20.0, Medcalc15.0 software, and R language were used for statistical analysis in the training set. Measurement data conforming to a normal distribution are expressed as the mean ± standard deviation ( $\bar{x}$ ± *s*), skewed data are expressed as the median (range), and the Mann–Whitney U test was used for comparisons between groups. Enumeration data were expressed as rates, and the χ^2^ test was applied. Logistic regression (started with all candidate variables with *P<*0.05 in the univariate logistic model) was performed to identify clinical and imaging features associated with malignancy. The model was trained on the whole dataset and tested in an independent test set. Using the pathological results as the gold standard, the sensitivity, specificity, and accuracy of the different diagnostic methods were calculated. Differences were considered statistically significant at *P<*0.05.

### Machine learning

#### Features

Twenty features were selected, which can be grouped into three groups. First, the clinical features were selected by the National Comprehensive Cancer Network (NCCN) guidelines (24). Second, the ultrasonic features were selected according to the BI-RADS lexicon (25). Third, the strain elastography and S-Detect results were extracted from the ultrasound machine.

#### Data pre-processing, feature selection, and model development

**Figure 1:**
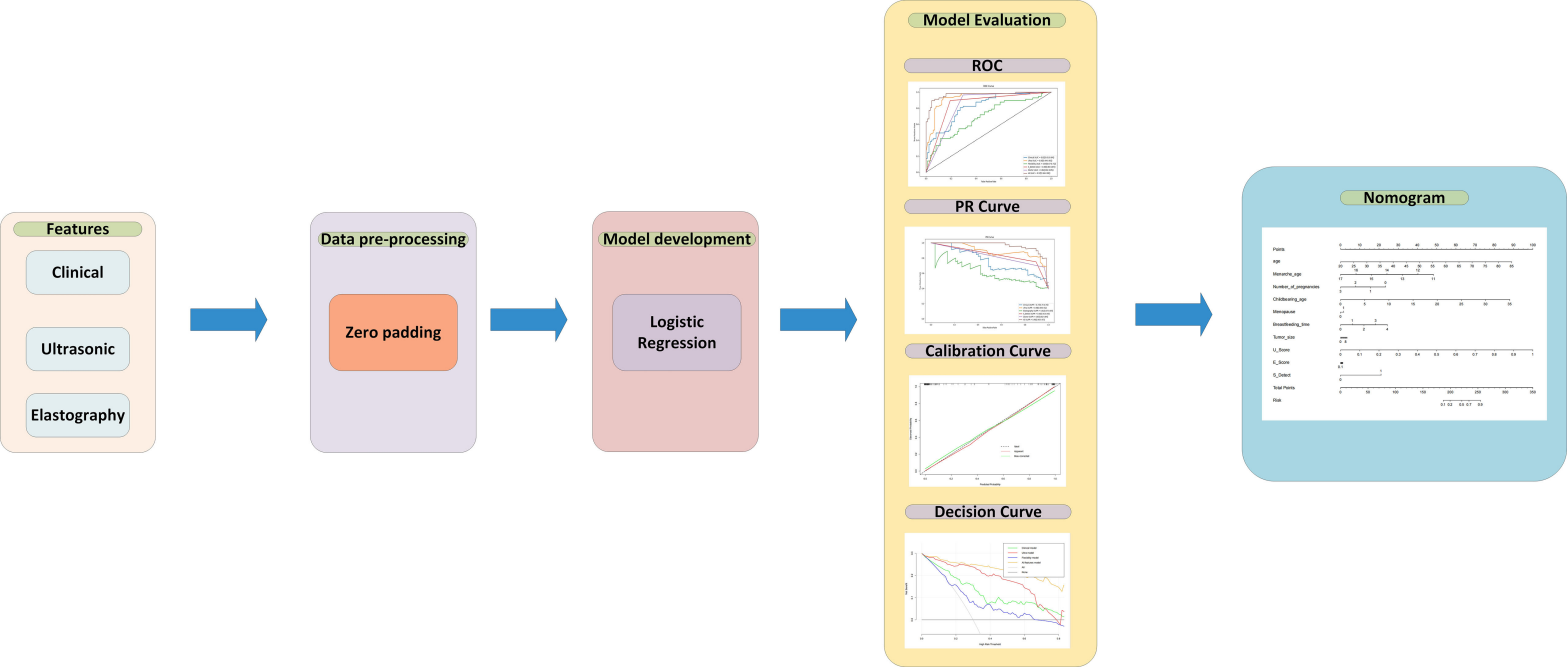
Schematic representation of the study flow. PR curve represent precision-recall curves.

For data preprocessing, we applied the zero-value method to pad the missing data in the training set (26). After feature selection, the logistic regression (LR) algorithm was applied as the backbone of the models. LR is one of the most common algorithms in the field of machine learning, which is often used as a baseline for processing binary classification tasks. The trained LR model will get the weights of each input feature and predict the possibility of malignant masses. In this article, four models were built: 1) the clinical model using only clinical features, 2) ultrasonic model with only parameters from the classic ultrasound scan, 3) the elastography model which features comes from elasticity score and elasticity ratio (Eratio and Ebreast), 4) combined all features model which uses clinical, ultrasound, elastography features and S-Detect result. Each model was built by the selected features to obtain the function and feature weights between the features and malignant breast masses. All four models were trained on the training set, which contained 145 patients, and 34 patients were used as an independent test set to verify the performances between different models. To evaluate the performance of the model, accuracy, recall, sensitivity, and specificity were calculated. Besides that, the ROC curve, precision-recall (RP) curve, calibration curve, and decision curve were plotted to illustrate the performance of the model. Using pathological results as the gold standard, the ROC curve and the AUC score for the S-Detect result and the doctors’ diagnosis were compared with the logistic regression model. In addition, the final nomogram model was built by combining the S-Detect result, the elastography model, the clinical model, and the ultrasound model. The model development, calibration curve, decision curve, and nomogram were built using the R language. The research flow is shown in **Figure 1**.

## Results

### Basic clinical information

A total of 189 breast lesions in 189 consecutive patients were included. Among them, 10 patients with 10 lesions were excluded, of which six patients presented six non-mass lesions on ultrasound and four had confined mastitis. Finally, a total of 179 breast lesions in 179 patients were included. The median age of the patients in this study was 50 years (range, 22–85 years). Among the 179 breast lesions, 101 (56.42%) were benign, while 78 (43.58%) were malignant. A total of 123 of 179 patients (68.72%) were asymptomatic, and 56 of 179 patients (31.28%) had palpable masses. A total of 137 masses were confirmed by core needle biopsy or surgical pathology. Forty-two lesions had typical benign features, and no significant changes were observed during the 2-year follow-up. The clinical characteristics and pathological results of the patients are shown in **Table 1**.

**Table 1:** Clinical information and pathological results of the patients.

Clinical information	Training set (n = 145)	Test set (n = 34)
Age (year)		
Median (range)	51 (22–85)	57 (27–82)
Menarche age (year)		
Median (range)	13 (11–17)	13 (12–15)
Number of pregnancies		
0	24	10
1	58	20
2	42	2
3	21	2
Age of primipara (year)		
Median (range)	24 (19–32)	27 (19–31)
Lactation time(month)		
<6 months	14	3
6–12 months	56	17
12–18 months	32	4
18–24 months	16	0
Menopause		
No	76	7
Yes	69	27
Tumor Size (cm)		
Median (range)	1.20 (0.24–10.0)	1.38 (0.57–4.13)
Pathologic findings		
Benign	88	13
Fibroadenoma	52	7
Adenopathy	27	3
Proliferative lesion	6	2
Intraductal papilloma	2	0
Hyaline change	1	1
Malignant	57	21
Invasive ductal carcinoma	29	14
Invasive lobular carcinoma	18	1
*In situ* ductal carcinoma	4	3
Solid papillary carcinoma	4	0
Mucinous carcinoma	1	1
Lymphoma	1	0
Malignant phyllodes tumor	0	2

### The outcomes of different models to predict and classify benign and malignant breast nodules

**Figure 2:**
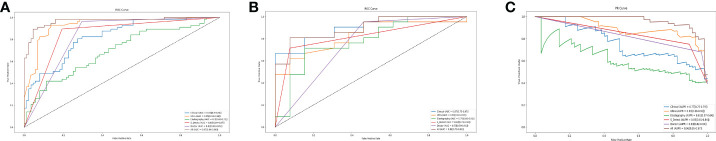
The ROC Curves in training set **(A)**. The ROC Curves **(B)** and the PR Curves **(C)** independent test set of the models based on the selected features. Clinical presents the model built with clinical features. Ultra presents the model built with ultrasonic features. Elastography presents the model built with elastography features. All present the model build with the combined features (clinical, ultrasonic, elastography, S-Detect).

The AI models constructed based on the selected features to predict and classify benign and malignant breast nodules were compared. The ROC curves in the training set and independent test set are plotted in **Figures 2A, B**. The AUCs of the models built on clinical features, ultrasonic features, and elastography features were 0.87 (95% CI: 0.75–0.97), 0.81 (95% CI: 0.61–0.93), and 0.79 (95% CI: 0.66–0.91), while the AUCs of the sonographer and S-Detect were 0.75 (95% CI: 0.59–0.91) and 0.82 (95% CI: 0.73–0.93), and the AUC of the AI model built on combined features (clinical, ultrasonic, elastography, and S-Detect) was 0.89 (95% CI: 0.79–0.96). The AUPR has similar trends in that the combined features model has the best performance of 0.96 (95% CI: 0.95–0.97) (**Figure 2C**). As also shown in **Table 2**, the AI model combining all of the features showed a relatively better specificity (SPE) (0.92 (95% CI: 0.82–1.0)) than the other models. The sonographer’s assessment showed better sensitivity (SEN) (0.97 (95% CI: 0.90–1.0)).

**Table 2:** The outcomes of different models to classify and predict benign and malignant breast nodules.

Models	Clinical	Ultrasound	Elastography	S_Detect	Doctor	All
AUC	0.87 (95%CI: 0.75–0.97)	0.81 (95%CI: 0.61–0.93)	0.79 (95%CI: 0.66–0.91)	0.82 (95%CI: 0.73–0.93)	0.75 (95%CI: 0.59–0.91)	0.89 (95%CI: 0.79–0.96)
ACC	0.75 (95%CI: 0.53–0.87)	0.72 (95%CI: 0.53–0.87)	0.60 (95%CI: 0.347–0.72)	0.78 (95%CI: 0.69–0.91)	0.81 (95%CI: 0.71–0.93)	0.75 (95%CI: 0.62–0.84)
SEN	0.64 (95%CI: 0.43–0.85)	0.59 (95%CI: 0.36–0.84)	0.45 (95%CI: 0.27–0.65)	0.70 (95%CI: 0.55–0.86)	0.97 (95%CI: 0.90–1.0)	0.65 (95%CI: 0.42–0.82)
SPE	0.93 (95%CI: 0.72–1.0)	0.94 (95%CI: 0.77–1.0)	0.88 (95%CI: 0.77–1.0)	0.94 (95%CI: 0.81–1.0)	0.53(95%CI: 0.22–0.84)	0.92 (95%CI: 0.82–1.0)

AUC, area under curve; ACC, accuracy; SEN, sensitivity; SPE, specificity.

### Visualization and clinical application of the AI model of all features

Based on the above results, the AI model of the combined features was selected. The ultrasound score (U score) and elastic score (E score) were calculated for application in the nomogram.

U Score =

0.034 ∗ Location + 2.636 ∗ shape + 2.246 ∗ boundary + 0.630 ∗ edge − 1.935 ∗ calcification + 1.468 ∗ Aspect_ratio + 1.233 ∗ Internal_echo − 2.026 ∗ attenuation + 1.121 ∗ Blood_flow − 3.67

E Score =

0.056 ∗ E_Strain + 0.637 ∗ E_Breast + 0.289 ∗ Elastic _score − 2.549.

**Figure 3:**
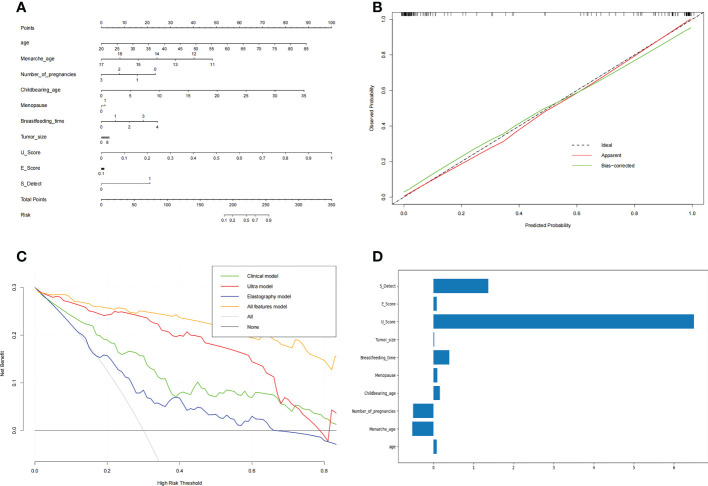
The nomogram of benign and malignant breast nodules **(A)**, the calibration curve of the nomogram **(B)** and the decision curve of different AI models to predict and classify benign and malignant breast nodules **(C)**. The features’ importance **(D)** was shown with the rate of weight distribution of the nomogram. **(A)** Nomogram was used to visualize the AI model for clinical application. The left side is the features age, menarche age, number of pregnancies, childbearing age, menopause, breastfeeding time, tumor size, U score, E score, and S detect that were used in the prediction model. The line segment corresponding to each variable is marked with a scale, which represents the range of possible values of the feature, and the length of the line segment reflects the contribution of the feature to the outcome event. Each feature has a corresponding point under different values. Add the points of all features together to get the total points of the patient. Based on the total points, draw a vertical line downward to know the risk for malignant breast nodules. **(B)** In the Calibration Curve, the abscissa represents the probability predicted by the nomogram, and the ordinate represents the actual probability of malignant breast nodules. A perfect prediction corresponds to the black dotted line. The solid red line represents the entire cohort, and the solid green line is bias-corrected by Bootstrapping (1,000 repetitions) and represents the observed nomogram performance. **(C)** The decision curve of the elastic model, clinical model, ultrasound model, and all-features model. The abscissa of this picture is the threshold probability. When various evaluation methods reach a certain value, the risk probability is recorded as Pi; when Pi reaches a certain threshold (referred to as Pt), it is defined as positive, and certain measures (such as predicting benign and malignant breast nodules) are taken. The balance of pros and cons then changes. The ordinate is the Net Benefit rate (NB) after subtracting the pros and cons. In addition to the curves of the four AI models, there are two gray lines in the figure. They represent two extreme cases. The horizontal bar indicates that all samples are negative (Pi<Pt), no intervening, and the NB is 0. The sloping line indicates that all samples were positive, all received the intervention, and the NB is a backslash with a negative slope. Other curves are compared with them. **(D)** The features importance of weight distribution of the nomogram. The U-score, ultrasound features, showed the highest impact, followed by S-Detect. Interestingly, age at menarche, number of pregnancies, and duration of breastfeeding also indicated important influences.

A nomogram (**Figure 3A**) was used to visualize the AI model for clinical application. The left side shows the age, menarche age, number of pregnancies, childbearing age, menopause, breastfeeding time, tumor size, U score, E score, and S-detect that were used in the prediction model. The line segment corresponding to each variable is marked with a scale, which represents the range of possible values of the feature, and the length of the line segment reflects the contribution of the feature to the outcome event. Each feature has a corresponding point under different values. The points of all features are summed up to obtain the total points of the patient. Based on the total points, a vertical line is drawn downward to determine the risk for malignant breast nodules.

In the calibration curve (**Figure 3B**) for the nomogram, the abscissa represents the probability predicted by the nomogram, and the ordinate represents the actual probability of malignant breast nodules. A perfect prediction corresponds to the black dotted line. The solid red line represents the entire cohort, and the solid green line is bias-corrected by bootstrapping (1,000 repetitions) and represents the observed nomogram performance. **Figure 3B** shows that the nomogram performance was comparable to a perfect prediction.

In regard to the clinical model and the radiomic model, we showed their decision curves in **Figure 3C**. The abscissa of this picture is the threshold probability. When various evaluation methods reach a certain value, the risk probability is recorded as Pi; when Pi reaches a certain threshold (referred to as Pt), it is defined as positive, and certain measures (such as predicting benign and malignant breast nodules) are taken. The balance of pros and cons then changes. The ordinate is the net benefit rate (NB) after subtracting the pros and cons. In addition to the curves of the four AI models, there are two gray lines in the figure. They represent two extreme cases. The horizontal bar indicates that all samples are negative (Pi<Pt), no intervention occurs, and the NB is 0. The sloping line indicates that all samples were positive, all received the intervention, and the NB is a backslash with a negative slope. The other curves were compared with them. **Figure 3C** shows that within a large Pt range, the benefits of the elastography model, clinical model, ultrasound model, and all-features models are higher than the extreme curves. Therefore, their optional Pt ranges are relatively large, which means they are relatively safe and effective. The Pt range of the all-feature model is relatively larger and better.

The features’ importance was shown by the rate of weight distribution of the nomogram (**Figure 3D**). The U-score (ultrasound features) showed the highest impact, followed by S-Detect. Interestingly, age at menarche, number of pregnancies, and duration of breastfeeding also indicated important influences.

## Discussion

Breast cancer is the most common malignant tumor in women (27). With the rapid development of computer technology, AI has shown good performance in diagnosis in the field of medical imaging. Recently, Wu et al. compared different deep-learning models based on the multi-input resolution for breast ultrasound images. They attempted to select the best DL combination (28). This study established an AI model with selected preoperative clinical features to improve the accuracy of the assessment of benign and malignant breast lesions.

Among the different diagnostic models in this study, the diagnostic model combining all features showed the highest diagnostic efficiency and was relatively better than the other models. The combined model considered the patient’s clinical information, the radiologist’s evaluation of the ultrasound images, ultrasound elastography, and the S-Detect results to form a comprehensive diagnosis. The AUC of the combined model was better than that of ultrasound, S-Detect, or elastography alone. This means that combined diagnosis is an important method to improve the differential diagnosis of benign and malignant breast lesions. Combined diagnosis can reduce the misdiagnosis rate and provide a more comprehensive and accurate image diagnosis basis for clinical practice. Previous studies have shown that the diagnostic performance of S-Detect moderate is consistent with that of a radiologist (kappa values = 0.58) (29). Park et al. (30) reported that S-Detect assistance could notably improve the AUC and interobserver agreement, especially for less experienced radiologists. Computer-aided diagnosis can serve as a “second opinion” to radiologists during morphological interpretation to improve their accuracy (31).

Our results showed that the combined AI diagnosis model could achieve complementary advantages, which are of great significance for the differential diagnosis of benign and malignant breast masses and can help doctors make better clinical decisions and reduce unnecessary biopsies. According to the weight map, U-Score and S-Detect seemed to be more important to distinguish benign and malignant breast masses in the combined model. The U-score (ultrasound features) showed the highest impact, followed by S-Detect. This may be related to the following factors. The U-score is a comprehensive score based on ultrasound features extracted by sonographers. The U-score includes nodule shape, aspect ratio, edge, capsule, internal echo, calcification, and color Doppler. The features covered by U-score are relatively more comprehensive than those observed by S-Detect. S-Detect technology (Samsung Healthcare, Seoul, South Korea) is a new artificial intelligence ultrasound-assisted diagnostic technology that uses a deep-learning algorithm and a convolutional neural network. Based on big data analysis using databases, it can provide a reference for the differential diagnosis of benign and malignant breast lesions. In terms of data sources, S-Detect data come from ultrasonic radio frequency time series. Compared to the grayscale image, there is no loss of information in radio frequency waves. In addition, S-Detect provides multiple segmentation modalities combined with ultrasound, so users can choose a more meticulous segmentation modality. S-Detect has a high stability and classification accuracy, which can reduce the operator dependence and the influence of subjective factors, and improve the conventional ultrasound diagnosis confidence (32). Especially in clinical diagnosis, more information could allow doctors to more accurately diagnose patients, which is the same with AI. The model with clinical, ultrasonic, elastography, and S-Detect information obtains the best performance in the four models. That illustrates, compared with the previous image-only method, the multi-modality of information can provide details to help AI diagnosis. Elastography is a noninvasive diagnostic method that can provide qualitative and quantitative information about the stiffness and elastic properties of tissue. Elastography as a supplement to conventional US can be used to distinguish benign and malignant breast lesions. A multicenter prospective study showed that the combination of S-Detect and elastography could further improve the diagnostic ability of US for asymptomatic breast nodules. Compared with a single use of S-Detector conventional ultrasound, S-Detect combined with elastography showed higher accuracy and specificity (21). Conversely, another study reported that the additional use of elastography did not show any improvement in the characterization of breast lesions compared to the use of morphology alone (22). Therefore, the value of elastography to assist diagnosis needs to be further explored. Many breast lesions are associated with microcalcifications. Certain types of microcalcifications are associated with negative genetic and molecular characteristics of the tumor and an unfavorable prognosis (22). S-Detect does not provide calcification information. Conventional ultrasound makes up for the deficiency of S-Detect in calcification information interpretation. S-Detect and traditional ultrasound can play a complementary role for each other in collecting diagnostic information.

Category 4 lesions based on the BI-RADS classification are defined as suspected malignancy, with malignancy rates ranging from 2% to 95% (25). Less experienced radiologists are at a greater risk of misdiagnosing cancer, increasing the number of false-positive diagnoses. Therefore, how to improve the diagnostic sensitivity of malignant lesions and reduce the puncture biopsy rate of benign lesions in clinical practice is of significant concern to radiologists. In this study, the specificity of the radiologist was 53% and that of the combined diagnosis was 92%; this improvement in specificity can reduce unnecessary biopsies, preserve medical resources, and reduce the psychological burden on patients. Previous studies have shown that the use of AI can lead to a change in the final BI-RADS classification, with a significant increase in the rate of correct reclassification and an improvement in final management decisions (29, 33, 34).

Nomograms are widely used for cancer prognosis and have the ability to reduce statistical predictive models into a single numerical estimate of the probability of an event, and they provide user-friendly graphical interfaces (35, 36). In this study, a nomogram was developed based on logistic regression analysis with ultrasound images, clinical information, ultrasound elastography, and S-Detect results. The area under the curve (AUC) was 0.89 in the training cohort. The decision curve derived from the nomogram displayed good clinical utility. This nomogram, as a noninvasive tool, provided a visual display of the diagnostic model, which can be used as a reference for doctors and may facilitate the development of more effective preoperative decision-making.

### Limitations

This study has some limitations that should be noted. First, this study was a single-center study, and non-mass lesions were not included. Second, only static images can be read with S-Detect (dynamic images cannot be read). However, the future development direction of artificial intelligence should not be limited to the analysis of static images; rather, dynamic videos should be collected to provide a strong basis for the extensive development of clinical ultrasound technology and to guide clinical diagnosis and treatment. Third, in this article, though the model with different features was compared, different algorithms with the same feature were not designed in the experiments. It is unclear whether there is an algorithm that suits this task better, and we will figure out it in future work. Fourth, in this article, the image features were extracted by radiomics, which means significant detail loss will appear in feature extraction processing compared to predicting directly from the image. In future work, we will combine the image and the clinical features into one CNN model for direct prediction. Fifth, the sample size of this study was small and was insufficient for the machine learning algorithm to sufficiently learn some important features within the data and provide a robust generalization ability of the developed AI models. A multicenter study with a large sample size should be performed to validate the performance of this AI model. We are going to develop more clinical and applicable software in the future.

## Conclusions

Elastic imaging can provide stiffness information about lesions. S-Detect can improve the classification of breast lesions in terms of diagnostic performance and operator dependence. Our experience suggests an AI model of sonographer’s evaluation + S-Detect + elastography + clinical information, with a higher overall AUC value and specificity than conventional US, could improve the preoperative identification of benign and malignant breast masses and may reduce the number of unnecessary breast biopsies.

## Data availability statement

The raw data supporting the conclusions of this article will be made available by the authors, without undue reservation.

## Author contributions

Study conception: PS, YF, JG, and ZW. Date collection: CC and PS. Date analysis: PS and ZW. Manuscript drafting: PS and YF. All authors contributed to the article and approved the submitted version.

## Funding

Capital’s Funds for Health Improvement and Research(NO.2022-4-1105).

## Conflict of interest

The authors declare that the research was conducted in the absence of any commercial or financial relationships that could be construed as a potential conflict of interest.

## Publisher’s note

All claims expressed in this article are solely those of the authors and do not necessarily represent those of their affiliated organizations, or those of the publisher, the editors and the reviewers. Any product that may be evaluated in this article, or claim that may be made by its manufacturer, is not guaranteed or endorsed by the publisher.
